# Supplementary material for: Phonon-enhanced nonlinearities in hexagonal boron nitride
Source: Nat Commun. 2023 Nov 24;14:7685. doi: 10.1038/s41467-023-43501-x (PMC10673846; doi:10.1038/s41467-023-43501-x)
Supplement: Supplementary file 1 — Supplementary Information [file 41467_2023_43501_MOESM1_ESM.pdf]

Supplementary Material For:
Phonon-Enhanced Nonlinearities in Hexagonal Boron Nitride

Jared S. Ginsberg[1,#,*], M. Mehdi Jadidi[1,#], Jin Zhang[2,#,*], Cecilia Y. Chen[3,#], Nicolas Tancogne-Dejean[2], Sang Hoon Chae[4,5,6], Gauri N. Patwardhan[1,7], Lede Xian[2], Kenji Watanabe[8], Takashi Taniguchi[9], James Hone[4], Angel Rubio[2,10,*], and Alexander L. Gaeta[1,3,*]

[1]*Department of Applied Physics and Applied Mathematics, Columbia University, New York, New York 10027, USA*
[2]*Max Planck Institute for Structure and Dynamics of Matter and Center for Free-Electron Laser Science, Hamburg 22761 Germany*
[3]*Department of Electrical Engineering, Columbia University, New York, New York 10027, USA*
[4]*Department of Mechanical Engineering, Columbia University, New York, New York 10027, USA*
[5]*School of Electrical and Electronic Engineering, Nanyang Technological University, Singapore 639798, Singapore*
[6]*School of Materials Science and Engineering, Nanyang Technological University, Singapore 639798, Singapore*
[7]*School of Applied and Engineering Physics, Cornell University, Ithaca, New York 14853, USA*
[8]*Research Center for Functional Materials, National Institute for Materials Science, 1-1 Namiki, Tsukuba 305-0044, Japan*
[9]*International Center for Materials Nanoarchitectonics, National Institute for Materials Science, 1-1 Namiki, Tsukuba 305-0044, Japan*
[10]*Center for Computational Quantum Physics, Simons Foundation Flatiron Institute, New York, NY 10010 USA*

[#]These authors contributed equally to this work
* jsg2208@columbia.edu *(J.S.G.)*, jin.zhang@mpsd.mpg.de *(J.Z.)*, angel.rubio@mpsd.mpg.de *(A.R.), and* alg2207@columbia.edu *(A.L.G.)*

Supplementary Discussion

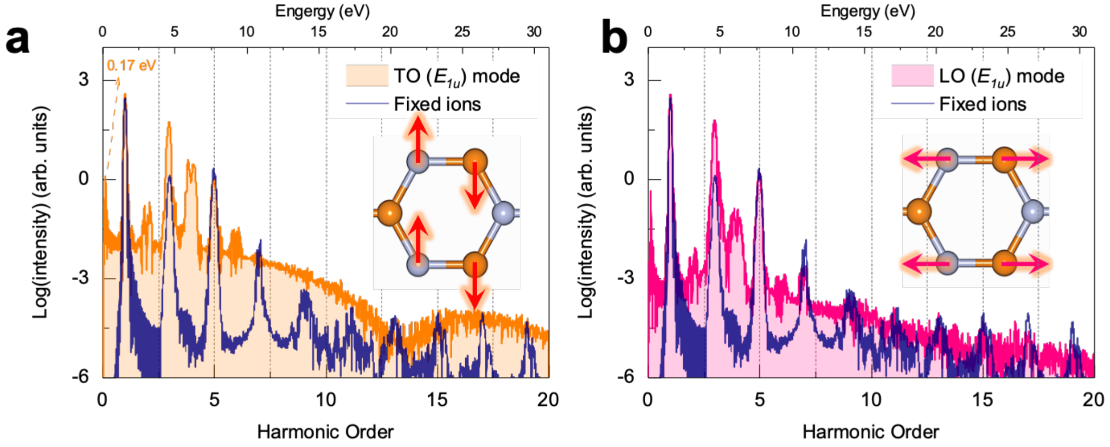

Figure S1: Calculated HHG spectra in the presence of an excited phonon mode, at two different pump laser polarizations. (a) Pump laser oriented parallel with the TO (E_{1u}) mode. (b) Pump laser oriented parallel with the LO (E_{1u}) mode. Here, we employ an in-plane electric field with a wavelength of $\lambda = 800$ nm and an intensity of $I = 10^{12}$ W /cm^2, and a pulse duration of 25-fs full width at half maximum. The phonon peak at 0.17 eV in panel (a) is highlighted for clarity.

HHG spectra obtained in the presence of the TO excited phonon display additional signals along with the odd harmonics. This results mostly from the presence phonon-induced sidebands, which are generated by two harmonic contents: i) the electron frequencies, which are the integer multiple of the laser field, and ii) the frequency of the phonon (ω_p). This can be explained using the following formula for HHG in solids[1].

$$HHG(\omega) \propto \left| FT\left(\int_{\Omega} dr\, n(r,t)\, \nabla v_0(r,t) \right) + N_e E \right|^2 , \quad (1)$$

where FT denotes the Fourier transform, Ω is the simulation cell volume containing N_e electrons, $n(r,t)$ is the time-dependent electronic density excited by the electric field E with frequency ω_0 and $v_0(r,t)$ is the electron-ion potential, which depends on time when phonons are excited. From this formula, the spectra, in presence of a phonon mode oscillating at frequency ω_p, contain a series of peaks at $(2n+1)\omega_0 + m\omega_p$.

From Figures S1, we note several smaller peaks, indicating high-order phonon-resonant processes are possible. However, we do not observe high-order signals since the strength of such processes are weaker than what our system can detect in the experiments. Operating with higher energy pulses to observe these processes resulted in damage to the sample.

The sideband effect also explains the dip at the even harmonic position in our simulations. The energy width between the two split peaks is approximately twice the energy of the TO mode, indicating that the nonlinearity is predominantly third-order. Figure S1a further confirms the importance of the phonon-induced oscillation since a peak is present in the spectrum at 0.17 eV, which corresponds to the energy of the vibrations of the TO (E_{1u}) mode in hBN (where electrons adiabatically follow the lattice). For a more general perspective, we explore the HHG spectrum with a driven longitudinal optical (LO) mode in Figure S1b. This demonstrates that the spectra contain mode-selective information.

It should be noted that we focus on the phonon mode at the Γ point because of 1) the energy E conservation between phonons and photons, i.e., $E = \hbar\Omega(q) = \hbar\omega(k)$, where $\Omega(q)$ is the phonon at the wave number q and $\hbar\omega(k)$ is the photon frequency at the photon wave number k; and 2) the momentum P conservation between phonons and photons, i.e., $\hbar k = \hbar q$. In our case, for the phonon momentum to satisfy both energy and momentum conservation, q should be very close to the Γ point. More specifically, the photon frequency $\omega(k)$ is the same as the phonon frequency $\Omega(q)$, and the photon momentum is $k \approx 1370\ cm^{-1}$, which is negligible compared to the size of the Brillouin zone where $|G| \approx 2 \times 10^8 cm^{-1}$, i.e., $\frac{k}{|G|} \approx 5 \times 10^{-6}$.

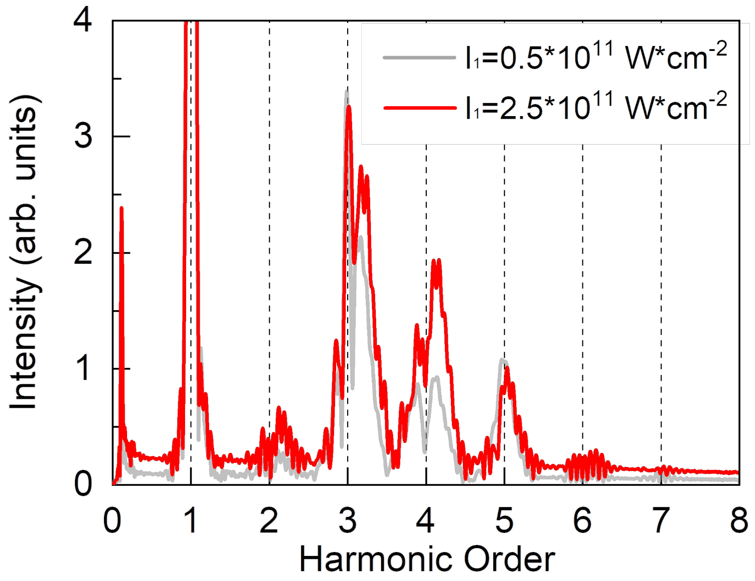

Figure S2: Calculated HHG spectra for different pump laser intensities in linear scale. Here, the polarizations of pump and probe laser are parallel with TO (E_{1u}) mode (pump laser with a wavelength of λ = 7300 nm). For the probe laser, we use an in-plane driving electric field with a wavelength of λ = 800 nm and an intensity of I = 10^{12} W/cm^2, and a pulse duration of 25-fs full width at half maximum.

The powers being modified in the two spectra of Figure S2 are the pump powers, while the harmonic signals are being generated by the probe pulse which is held constant at 10^{12} W/cm^2. The odd-order harmonics are almost entirely probe power dependent (not pump-mediated). In the two spectra above with pump intensities of 0.5x10^{11} W/cm^2 and 2.5×10^{11} W/cm^2, the 3rd and 5th harmonic intensities increased by factors of 1.5 and 1.0, respectively. By comparison, the novel sideband effects are driven by the pump power, and we observe a relatively larger 4.3-times enhancement of the signal near the second harmonic frequency.

Supplementary Note 1

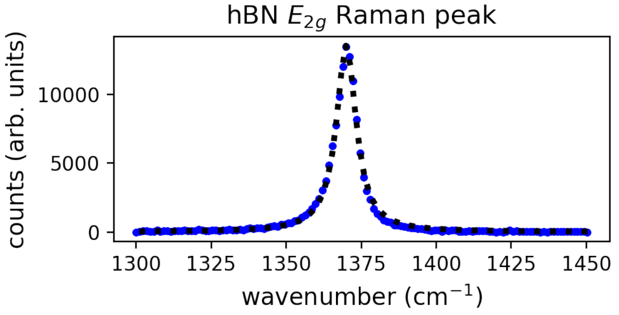

Figure S3: Raman spectrum of the hBN E_{2g} peak.

The Raman spectrum corresponding to the E_{2g} phonon mode of the hBN sample is included in Figure S3. The infrared-active phonon mode of interest at 7.3 um is characterized by a linear reflection spectrum about

the resonance, taken via Fourier-transform infrared (FTIR) spectroscopy[2].

Supplementary Note 2

From Figure 5a, the peak THG intensity for a pump wavelength of 7.3 μm is 16.6 times greater than the minimum measurable off-resonance THG signal (corresponding to a pump wavelength of 6.9 μm). To our knowledge, measurements of $\chi^{(3)}$ in hBN have not been performed in the mid-IR. Popkova et al. reported a third-order susceptibility value of 8.4 x 10^{-21} m^2/V^2 for a wavelength of 1080 nm in the near-infrared[3]. Using this value, we can extrapolate an order-of-magnitude estimate for the lower bound of $\chi^{(3)}$ on resonance as 10^{-19} m^2/V^2.

Supplementary References

1. Tancogne-Dejean, N., Mücke, O. D., Kärtner, F. X. & Rubio, A. Impact of the Electronic Band Structure in High-Harmonic Generation Spectra of Solids. *Physical Review Letters* **118**, 087403 (2017).
2. Chen, C. Y. *et al.* Unzipping hBN with ultrashort mid-infrared pulses. Preprint at https://doi.org/10.48550/arXiv.2205.12310 (2022).
3. Popkova, A. A. *et al.* Optical Third-Harmonic Generation in Hexagonal Boron Nitride Thin Films. *ACS Photonics* **8**, 824–831 (2021).
